# The complete chloroplast genome of Tibetan medicinal plant *Rubus phoenicolasius* Maxim

**DOI:** 10.1080/23802359.2021.1886013

**Published:** 2021-03-15

**Authors:** Guoying Zhang, Yarong Liu, Ping Hai

**Affiliations:** Qinghai Provincial Key Laboratory of Modernization of Traditional Chinese and Tibetan Medicine, Qinghai Provincial Drug Inspection and Testing Institute, Xining, China

**Keywords:** *Rubus phoenicolasius*, chloroplast genome, phylogenetic analysis

## Abstract

*Rubus phoenicolasius* Maxim. is a traditional Tibetan medicine and widely used in the clinical pharmacology. In current study, the complete chloroplast genome of *R. phoenicolasius* was reported. The total length of the genome was 155,144 bp with the GC content of 37.9%. We predicted 130 genes in the genome including 84 protein-coding genes, 37 tRNA genes, 8 rRNA genes and 1 pseudogene. 17 genes were duplicated in the IR regions including 7 tRNA, 4 rRNA and 6 protein-coding genes. Phylogenomic analysis revealed that *R. phoenicolasius* forms a strong supported branch with *R. amabilis* and *R. coreanus* under the Rosaceae clade.

*Rubus phoenicolasius* Maxim. (urn:lsid:ipni.org:names:895791-1), belonging to Rosaceae, is mainly distributed in China, Japan and Korea, and is naturalized in Europe and North America (Wu et al. 2003). As a traditional Tibetan medicine, *R. phoenicolasius* has long been used for the treatment of rheumatism, irregular menstruation and kidney ailments (Yang 1991). In recent years, most research concerning *R. phoenicolasius* mainly focused on its chemical composition (Luo et al. 2014), pharmacological activity (Liu et al. 2014), genetic diversity (Innis et al. 2011), and little is known about the chloroplast genome of *R. phoenicolasius*. In the current study, we report the complete chloroplast genome of *R. phoenicolasius* based on the next-generation sequencing method.

A wild individual of *R. phoenicolasius* was collected from Qinghai, China (N 32°39′33″, E 100°57′35″). The specimen was deposited at Qinghai-Tibetan Plateau Museum of Biology (HNWP, Xiaofeng Chi, xfchi@nwipb.cas.cn) under the voucher number Chi202002. The total genomic DNA was extracted from the fresh leaves with the modified CTAB method (Doyle 1987). The DNA sample was randomly fragmented to construct paired-end libraries according to the Illumina standard protocol and the VAHTS Universal DNA Library Prep Kit (Vazyme Biotech, Nanjing, China) was used for preparing the library. Sequencing was conducted on the Illumina Novaseq platform (San Diego, CA, USA). The Q20 and Q30 of the clean data were 97.65% and 92.92%, respectively, which showed that the quality of sequencing was fine. The complete chloroplast genome was assembled by SPAdes v3.10.1 (Bankevich et al. 2012) with the kmer setting at 55, 87 and 121. Annotation was performed on CPGAVAS2 (Shi et al. 2019) coupled with manual adjustment of start/stop codons and intron/exon borders after BLAST searches.

The total length of the *R. phoenicolasius* chloroplast genome was 155,144 bp including two inverted repeats (IR, 25,973 bp), a large single-copy region (LSC, 84,618 bp) and a small single-copy region (SSC, 18,580 bp). The GC content of the genome is 37.9% with the LSC of 35.6%, the IR of 42.9% and SSC of 31.9%. 130 genes was predicted, including 84 protein-coding genes, 37 tRNA genes, 8 rRNA genes and 1 pseudogene (ycf1). Seven tRNA, four rRNA and six protein-coding genes were duplicated in the IR regions.

Phylogenetic analysis was performed on the PhyloSuite platform (Zhang et al. 2020). The whole plastome sequences of *R. phoenicolasius* and 28 species in Rosaceae was used for the analysis. Meanwhile *Ulmus gaussenii* (Ulmaceae) was used as the outgroup. Sequences were aligned using MAFFT (Kazutaka and Standley 2013), and ModelFinder was employed to select the best model for phylogenetic analysis. A maximum likelihood (ML) analysis was conducted on IQ-TREE based on the TVM + F+R3 nucleotide substitution model with 1000 replications (Figure 1). The phylogenetic tree showed that *R. phoenicolasius* forms a strong supported branch with *R. amabilis* and *R. coreanus* under the Rosaceae clade. We expect that the chloroplast genome of *R. phoenicolasius* will be a valuable resource for future studies on conservation genetics, taxonomy, and phylogeny involving this particular species.

**Figure 1.**
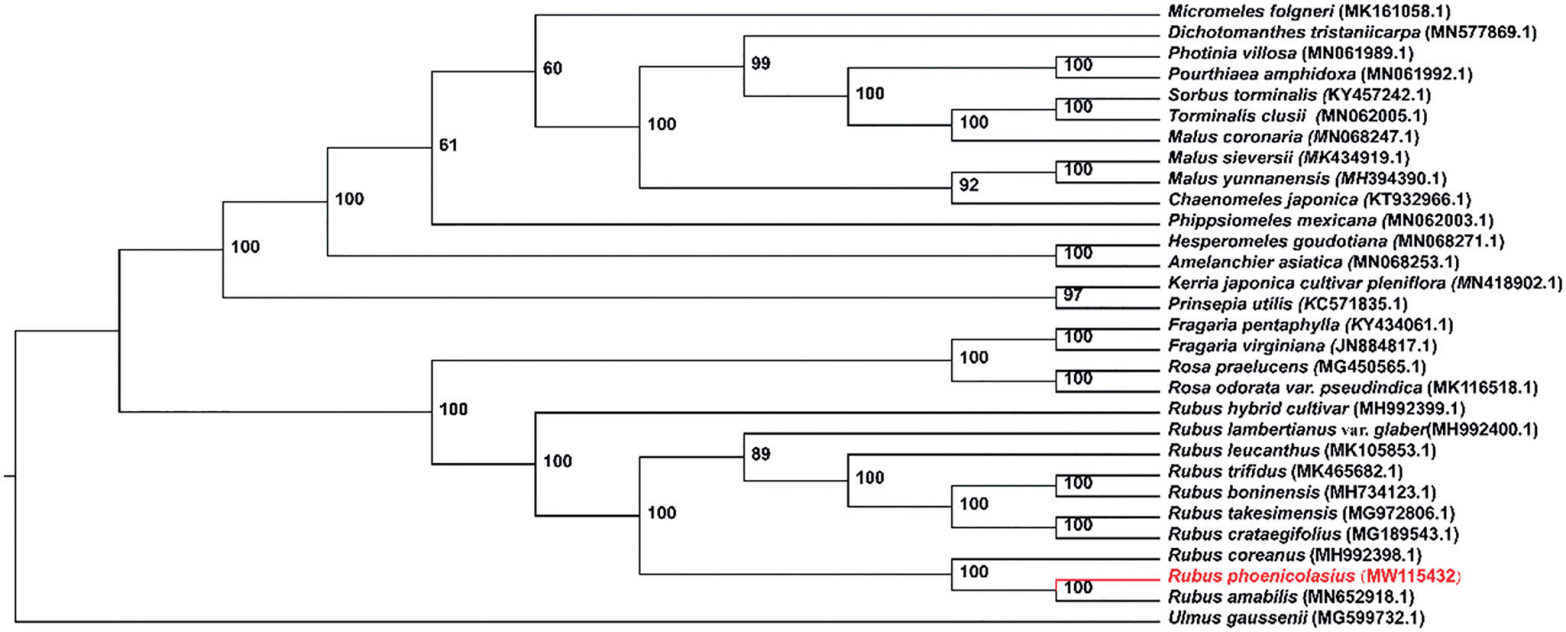
IQ-TREE based on 30 complete chloroplast genome sequences. Numbers associated with the branches are bootstrap values.

## Data Availability

The chloroplast genome and raw sequencing data in this study are available in GenBank (https://www.ncbi.nlm.nih.gov/) under the accession numbers of MW115432 and SRR13021958.
